# Bioavailability and pharmacokinetics of oral meloxicam in llamas

**DOI:** 10.1186/1746-6148-8-85

**Published:** 2012-06-21

**Authors:** Amanda J Kreuder, Johann F Coetzee, Larry W Wulf, Jennifer A Schleining, Butch KuKanich, Lori L Layman, Paul J Plummer

**Affiliations:** 1 Department of Veterinary Diagnostic and Production Animal Medicine (VDPAM), College of Veterinary Medicine, Iowa State University, Ames, IA, 50011, USA; 2 Department of Veterinary Microbiology and Preventative Medicine (VMPM), College of Veterinary Medicine, Iowa State University, Ames, IA, 50011, USA; 3 Cyclone Custom Analyte Detection Service (CYCADS), College of Veterinary Medicine, Iowa State University, Ames, IA, 50011, USA; 4 Department of Veterinary Clinical Sciences, College of Veterinary Medicine, Iowa State University, Ames, IA, 50011, USA; 5 Department of Anatomy and Physiology, College of Veterinary Medicine, Kansas State University, Manhattan, KS, 66506, USA

**Keywords:** Camelid, Llama, Pharmacokinetics, Meloxicam, NSAIDS, Oral bioavailability

## Abstract

**Background:**

South American camelids in the United States have rapidly developed into an important agricultural industry in need of veterinary services. Pain management is challenging in camelids because there are no drugs currently approved by the U.S. Food and Drug Administration for use in these species. Dosage regimens used for many therapeutic drugs have been extrapolated from other ruminants; however, the pharmacokinetics, in camelids, may differ from those of other species. Studies investigating the pharmacokinetics of cyclooxygenase-2 (COX-2) selective non-steroidal anti-inflammatory drugs in camelids are deficient in the published literature. Six adult llamas (121- 168 kg) were administered either a 1 mg/kg dose of oral or a 0.5 mg/kg dose of IV meloxicam in a randomized cross-over design with an 11 day washout period between treatments. Plasma samples collected up to 96 hours post-administration were analyzed by high pressure liquid chromatography and mass spectrometry detection (HPLC-MS) followed by non-compartmental pharmacokinetic analysis.

**Results:**

A mean peak plasma concentration (C_MAX_) of 1.314 μg/mL (Range: 0.826 – 1.776 μg/mL) was recorded at 21.4 hours (Range: 12.0 – 24.0 hours) with a half-life (T ½ λ_z_) of 22.7 hours (Range: 18.0 – 30.8 hours) after oral meloxicam administration. In comparison, a half-life (T ½ λ_z_) of 17.4 hours (Range: 16.2 – 20.7 hours) was demonstrated with IV meloxicam administration. The oral bioavailability (F) of meloxicam (dose normalized) was 76% (Range: 48 – 92%). No adverse effects associated with either treatment modality were observed in the llamas.

**Conclusions:**

The mean bioavailability (F) of oral meloxicam was 76% indicating a high degree of gastrointestinal absorption. Plasma meloxicam concentrations >0.2 μg/mL were maintained for up to 72 h after oral administration; >0.2 μg/mL is considered to be the concentration of meloxicam required for analgesic effects in other species such as the horse. These data suggest that a single dosage of oral meloxicam at 1 mg/kg could potentially maintain therapeutic concentrations in plasma for up to 3 days in adult llamas.

## Background

Over the past few decades, North America has seen a rapid increase in the number of South American camelids raised for fiber, show and companionship. Along with the increase in animal numbers has come an increase in interest for veterinary care and appropriate drug therapies for these animals. To date, however, there are no drugs currently approved by the Food and Drug Administration for use in camelids in the United States. Thus, extra-label use of common veterinary therapeutic drugs in camelids is routine in practice, yet there generally remains a lack of pharmacokinetic (PK) data in camelids. Dosages used have been extrapolated from other large animal species, including cattle, horses, and other small ruminant species. Particularly in regards to oral administration and bioavailability of medications, this practice has proven to be inadequate in some of the camelid pharmacokinetic studies published to date [1-5]. In addition, although not common practice in the United States, llama meat is available for purchase through various outlets. If this market were to expand, scientific data on drug withdrawal time in llamas are currently lacking to ensure the safety of this non-traditional food supply.

Non-steroidal anti-inflammatory drugs (NSAIDS) are commonly utilized in large animal veterinary practice for relief of pain, fever, and inflammation. Increasing public awareness of animal welfare will likely continue to make proper use of NSAIDS a priority in the treatment of all domestic animals for painful inflammatory conditions. Therefore, knowledge of the pharmacokinetics of NSAIDS in the specific species of interest will be required to provide for safe and efficacious use. Previous studies have been completed evaluating the pharmacokinetics of the NSAIDS flunixin meglumine [6], ketoprofen [7], and phenylbutazone [8] in llamas, however, studies investigating the pharmacokinetics, in camelids, of non-steroidal anti-inflammatory drugs considered to be cyclooxygenase-2 (COX-2) selective are deficient in the published literature. In general, the beneficial therapeutic actions of NSAIDS are thought to be related to inhibition of COX-2, and the undesirable side effects such as gastrointestinal ulceration due to non-selective inhibition of both COX isoforms[9]. Thus, the ability to utilize a COX-2 selective NSAID in a field setting for management of pain and inflammation may be desirable.

Meloxicam is an NSAID of the oxicam class which exerts its effect via selective inhibition of the COX-2 enzyme, thereby preventing prostaglandin synthesis which can lead to pain, fever and inflammation. Meloxicam is approved for use in cattle in the European Union (EU) as adjunctive therapy for acute respiratory disease, diarrhea and acute mastitis [10], and in Canada for alleviation of pain at debudding and improved performance in calves with diarrhea[11]. In companion animals meloxicam is approved in the EU, United States, and Canada for use in dogs for treatment of pain associated with osteoarthritis and cats for control of perioperative pain [12]. In addition, meloxicam has been shown in cattle to effectively suppress the inflammatory response to experimental endotoxin administration [13]. The availability of an oral formulation of meloxicam makes it particularly attractive for field use in treatment of inflammatory conditions.

As the pharmacokinetics of meloxicam in llamas have not been reported to date in the published literature, the objective of this study was to evaluate the pharmacokinetics of IV and oral meloxicam administration, and from this data determine the oral bioavailability in llamas. If oral meloxicam results in plasma concentrations comparable to that of parenteral administration, it may provide a practical and cost-effective method for relief of pain and inflammation in llamas.

## Results

**Figure 1:**
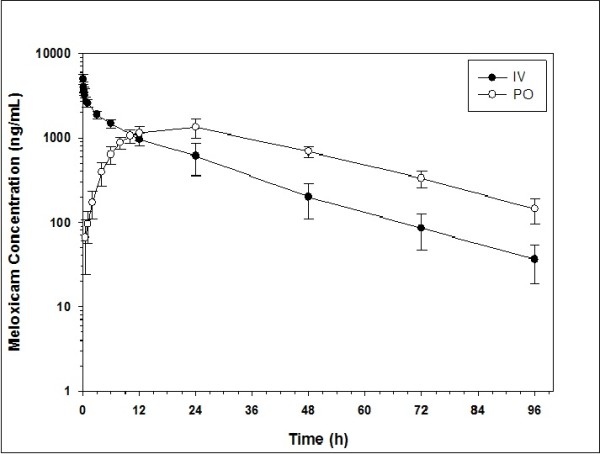
Plasma meloxicam concentrations in llamas after intravenous (IV) administration at 0.5 mg/kg and oral (PO) administration at 1 mg/kg.

No adverse effects were noted after IV or oral meloxicam administration. The pharmacokinetic parameters of meloxicam in llamas following IV administration of 0.5 mg/kg and oral administration at 1 mg/kg are shown in Table 1 and Figure 1. Following IV administration, meloxicam demonstrated a moderately small mean volume of distribution (Vss) of 0.235 L/kg (Range: 0.206 – 0.237 L/kg) and a relatively slow mean clearance (Cl) from the central compartment of 0.19 ml/min/kg (Range: 0.151 – 0.192 mL/kg/min). This was associated with a long mean plasma half-life (T ½ λ_z_) of 17.4 hours (Range: 16.2 – 20.7 hours).

**Table 1:** Meloxicam pharmacokinetic parameters following a single IV (0.5 mg/kg) or PO (1.0 mg/kg) administration

	**IV**				**PO**			
**Parameter (Units)**	**Geometric Mean**	**Min**	**Median**	**Max**	**Geometric Mean**	**Min**	**Median**	**Max**
**AUC**_**extrapolated**_**(%)**	1.9	1.4	1.7	3.5	6.6	3.6	6.3	13.1
**AUC**_**INF**_(hr*μg/mL)	43.96	31.44	43.45	55.60	68.35	52.37	74.98	79.24
**Cl (mL/min/kg)**	0.190	0.151	0.192	0.260	N/A	N/A	N/A	N/A
**Cl/F (mL/min/kg)**	N/A	N/A	N/A	N/A	0.248	0.212	0.230	0.318
**C0 (μg/mL)**	6.163	5.257	5.930	7.631	N/A	N/A	N/A	N/A
**C**_**MAX**_**(μg/mL)**	N/A	N/A	N/A	N/A	1.314	.826	1.368	1.776
**T**_**MAX**_**(hr)**	N/A	N/A	N/A	N/A	21.4	12.0	24.0	24.0
**T ½ λz (hr)**	17.4	16.2	16.8	20.7	22.7	18.0	21.9	30.8
**λz (1/hr)**	0.0398	0.0335	0.0413	0.0428	0.0306	0.0225	0.0316	0.0386
**MRT (hr)**	20.6	17.7	20.0	26.5	41.7	35.4	40.7	51.3
**Vss (L/kg)**	0.235	0.206	0.237	0.282	N/A	N/A	N/A	N/A
**Vz (L/kg)**	0.286	0.213	0.293	0.365	N/A	N/A	N/A	N/A
**Vz/F (L/kg)**	N/A	N/A	N/A	N/A	0.487	0.331	0.464	0.748
**Dose (mg/kg)**	0.50	0.49	0.50	0.51	1.0	1.0	1.0	1.1
**MAT (hr)**	N/A	N/A	N/A	N/A	20.8	13.9	22.6	24.8
**F**	N/A	N/A	N/A	N/A	0.76	0.48	0.85	0.92

Meloxicam noncompartmental pharmacokinetics (WinNonlin 5.2, Pharsight Inc. Cary NC, USA).

AUC extrapolated = percent of the AUC extrapolated; AUC_INF_ = area under the curve extrapolated to infinity; Cl = plasma clearance; Cl/F = Cl per fraction of the dose absorbed; C0 = Concentration extrapolated to time 0 using log-linear regression of the first two time points; T_MAX_ = time to C_MAX;_ T ½ λz = terminal half-life; λz = terminal rate constant; MRT = mean residence time extrapolated to infinity; Vss = volume of distribution at steady state; Vz = volume of distribution, area method; Vz/F = Vz per fraction of the dose absorbed; MAT = mean absorption time; F = fraction of the dose absorbed.

A mean peak plasma meloxicam concentration (C_MAX_) of 1.314 μg/mL (range 0.826 – 1.776 μg/mL) was recorded at approximately 21.4 hours (range 12.0 – 24.0 hours) following oral administration. This was associated with a mean plasma half-life (T ½ λ_z_) of 22.7 hours (Range: 18 – 30.8 hours) which is extended when compared to the T ½ λ_z_ observed with IV administration. The area under the curve extrapolated to infinity (AUC_INF_) following oral administration relative to IV administration resulted in a dose normalized calculated fraction of the drug absorbed (F) being 0.76 (range 0.48 – 0.92).

Table 2 provides a summary of the hematological parameters measured prior to and after the second phase of treatment with meloxicam (n = 3 for both oral and IV treatments). A reference range has not been established for this species at this lab; therefore standard reference ranges from Oregon State University Diagnostic Lab [14] were utilized by our laboratory to evaluate results. Some minor abnormalities in measured blood parameters were noted, however, all were considered to be of minimal clinical significance.

**Table 2:** Results of complete blood count and serum chemistries pre- and post- meloxicam treatment

		**Pre-tx**	**Post-tx**
**Parameter**	**Reference**	**Mean**	**Mean**
**(Units)**	**Ranges**†	**(min – max)**	**(min – max)**
**Sodium (mEq/L)**	146 – 156	153.83 (149–156)	151.17 (149–153)
**Chloride (mEq/L)**	109 – 125	115 (111–118)	118.83* (118–120)
**Potassium (mEq/L)**	3.8 – 7.3	4.13 (3.8-4.4)	4.35 (4.0-4.5)
**Calcium (mg/dL)**	8.4 – 10.8	9.2 (8.7-9.7)	9.3 (8.7-10.0)
**Bicarbonate (mEq/L)**	19 – 29	29.67 (27–33) ^a^	22.67* (22–24) ^a^
**BUN (mg/dL)**	24 – 44	15.00 (10–20) ^b^	21.17* (17–25) ^b^
**Creatinine (mg/dL)**	1.5 – 2.7	1.8 (1.7-2.0)	1.43** (1.3-1.5) ^a^
**Total Protein (gm/dL)**	5.3 – 7.3	5.83 (5.3-6.2)	5.87 (5.4-6.1)
**Albumin (gm/dL)**	3.0 – 4.2	2.67 (2.2-2.9) ^b^	2.85 (2.4-3.3) ^b^
**GGT (IU/L)**	27 – 78	41.83 (31–55)	49.83 (33–89) ^a^
**AST (IU/L)**	66 – 235	342.67 (156–1150) ^a^	319 (146–926) ^a^
**ALP (IU/L)**	12 – 97	49.17 (36–75)	55.83 (39–82)
**Total bilirubin (mg/dL)**	0.0 – 0.3	<.1	<.1
**Triglycerides (mg/dL)**	6 – 43	21.67 (17–25)	18.83 (10–26)
**NEFA (mmol/L)**	0 – 0.6 ‡	0.09 (0.01-0.21)	0.04* (0.01-0.08)
**WBC (x10^3/μL)**	8.0 – 21.4	9.89 (4.06-17.45) ^a^	9.84 (5.39-14.44) ^a^
**RBC (x10^6/μL)**	10.1 – 17.3	9.47 (8.16-10.9) ^a^	9.40 (7.97-10.69) ^a^
**PCV (%)**	27 – 45	25.25 (21.5-28) ^a^	24.67 (21.5-28) ^a^
**Fibrinogen (mg/dL)**	100 – 500	283 (200–400)	316 (200–500)

* = p value <0.05 for change in values between pre- and post-treatment samples.

** = p value <0.01 for change in values between pre- and post-treatment samples.

^a^ = results contain values outside of reference ranges.

^b^ = results for all animals were outside of reference ranges.

† = adult llama reference ranges obtained from the Clinical Pathology Laboratory, College of Veterinary Medicine, Oregon State University [14].

‡ = normal range not provided from lab, utilized range from Tornquist et al 1999.

## Discussion

An ideal anti-inflammatory drug for use in field settings requires the ability for oral administration, does not require multiple doses per day, minimizes the number of administration periods necessary to achieve adequate drug concentrations, and is cost-effective. The purpose of this study was to investigate the pharmacokinetics and oral bioavailability of meloxicam in llamas. The results of this study indicate that a mean C_MAX_ of 1.314 μg/mL occurred approximately 21.4 hours after oral administration. A relatively slow clearance resulted in a T ½ λ_z_ of 22.7 hours. Oral meloxicam demonstrated a high degree of gastrointestinal absorption and oral bioavailability when dose normalized in comparison to IV administration. These findings suggest that oral meloxicam may provide an effective means of providing long-lasting analgesic and anti-inflammatory effects to llamas in a field setting once efficacy has been demonstrated.

During the study, one of the llamas exhibited a large increase in weight (from 142.7 kg to 164.1 kg) between the first treatment (IV) and the second treatment (oral). This increase is considered to be too large for normal weight gain over an 11 day period (approximately 2 kg/day), thus error in weighing the animal was likely the source of variation. Previous hospital records of this animal indicate a weight of 172.7 kg approximately one month prior to initiation of the trial. Therefore, it is highly likely that the weight for the first treatment was recorded as falsely low at 142.7 kg and that the weight for the second treatment at 164.1 kg (which was verified with a second weighing at the time of the discovery of the discrepancy) is the more correct weight of the animal. This animal did consistently exhibit the lowest concentration for all time points when compared to IV administrations in all other animals, which suggests that the IV dosage administered may have been less than the targeted 0.5 mg/kg (calculated as 0.43 mg/kg based on the second weight). However, the variation in the data was minimal, and there was found to be no statistically significant difference between the calculated mean pharmacokinetic parameters whether data from this animal was included or not included in the final calculations. Additionally, as the incorrect weight was determined to be that used for IV administration, this minor discrepancy should not affect the overall evaluation of the data, especially in regards to oral bioavailability and pharmacokinetics.

As venous access in llamas is challenging and is generally reserved for direct use by veterinarians, oral administration is the preferred route over intravenous administration in field settings. Table 3 demonstrates the differences in the pharmacokinetic parameters in llamas of the NSAIDS that have been reported in the literature to date: meloxicam (present study), flunixin meglumine [6], ketoprofen [7], and phenylbutazone [8]. Of the four, PK values for oral administration have only been reported for phenylbutazone previously, and now meloxicam, while IV data is available for all four compounds studied. In comparison to oral administration of phenylbutazone, oral meloxicam exhibits similar AUC_INF_ and oral bioavailability but reaches T_MAX_ much later (21.4 hrs vs 4.4 hrs) and also possesses a much longer half-life (17.9 hrs vs. 7.1 hrs) even though plasma drug concentrations are detected within 30 minutes of administration. Therefore, oral administration of phenylbutazone could potentially provide therapeutic effects more rapidly than meloxicam, however, may not reach plasma levels considered to be therapeutic when compared to those reported in equine pharmacodynamic studies (EC50 ranging from 3.6 μg/mL to 15 μg/mL) [8,15,16]. The pharmacokinetic parameters of phenylbutazone also suggest a much shorter duration of effect when compared to meloxicam while requiring more frequent dosing with extended duration dosing regimens. In addition to the potential for oral administration, the ideal NSAID should not require multiple doses per day and should minimize the overall number of administration periods necessary to achieve adequate levels of drug concentration. In this regard, oral meloxicam offers a more desirable pharmacokinetic profile over oral phenylbutazone for longer term NSAID therapy in llamas.

**Table 3:** Comparison of pharmacokinetic parameters of common NSAIDS in llamas

	**IV dosages (mean values)**						**PO dosages (mean values)**	
**Parameter (Units)**	**Meloxicam (present study)**	**Phenylbutazone**[8]	**Ketoprofen**[7]*			**Flunixin meglumine**[6]	**Meloxicam (present study)**	**Phenylbutazone**[8]
			**S**	**R**	**Sum**			
AUC (hr*μg/mL)	43.96	80.1	168.9	176.4	345.3	49.0	68.35	60.8
**Cl (mL/min/kg)**	0.190	1.11	0.22	0.21	0.43	0.78	N/A	N/A
**T**_**MAX**_**(hr)**	N/A	N/A	N/A			N/A	21.4	4.37
**T ½ λz(hr)**	17.4	2.03	5.49	5.41	5.45	1.47	22.7	7.09
**Vss(L/kg)**	0.235	0.155	0.100	0.095	0.195	0.030	N/A	N/A
**Dose (mg/kg)**	0.50	5.0	2.2	2.2	4.4	2.2	1.0	5
**F (%)**	N/A	N/A		N/A		N/A	76.0	69.9

* For the ketoprofen study, a racemic mixture of S and R enantomers were administered to each animal. PK values from each enantomer as well as the sum (or in the case of half –life, average) of both are reported here.

AUC = area under the curve extrapolated to infinity; Cl = plasma clearance; T_MAX;_ = time to C_MAX;_ T ½ λz = terminal half-life; Vss = volume of distribution at steady state; F = fraction of the dose absorbed.

When administered IV, T ½ λ_z_ of meloxicam is substantially greater than in other NSAIDS studied thus far (17.6 hrs vs. 1.5–5.5 hrs); this is directly related to a slower clearance rate of meloxicam when compared to the previously studied NSAIDS (0.19 vs. 0.43–1 .11 mL/min/kg). Volume of distribution was also greater with meloxicam than with the other NSAIDS (0.235 vs. 0.03–0.195 L/kg), although all values can be considered to be within the range of a low volume of distribution. Most NSAIDS exhibit a high degree of protein binding which causes relatively low volume of distribution into the interstitial fluids but facilitates passage into areas of inflammation with leakage of plasma proteins into exudate [17]. These data suggest that if IV administration is chosen over oral, IV meloxicam will still provide a longer duration of activity than all other NSAIDS studied thus far in llamas. Interestingly, the finding of a longer T ½ λ_z_ following oral versus IV meloxicam administration in llamas is similar to the results seen in other ruminant or pseudo-ruminant species studied thus far (cattle, sheep, and goats) [18]. The elimination half-life is determined by the clearance and the volume of distribution of a drug and is typically the same irrespective of the route of administration. However, in cases where the rate of elimination is faster than the rate of absorption, the terminal slope is not parallel after oral administration because this reflects the absorption half-life rather than the elimination half-life. This is an example of “flip-flop” kinetics and has also been described after oral administration of meloxicam in other ruminants most likely because the rumen delays absorption of the drug from the gastrointestinal tract [18].

The pharmacokinetic parameters of most NSAIDS have been demonstrated to be species dependent, and meloxicam has proven to be no exception. Tables 4 and 5 list the differences in pharmacokinetic parameters of meloxicam in various domesticated large animal species [18-22]. The most closely related species studied thus far has been camels, however, only IV administration was evaluated in that study. The half-life of oral meloxicam in llamas (22.7 hrs) appears to be similar to species such as cattle (27.5 hrs) [18] and cats (24 hrs) [23] but is much slower than in other species such as goats (11.8 hrs) [19] and dogs (12.1 hrs) [24]. In contrast, T_MAX_ exhibits extreme variation, with llamas taking the longest time to reach maximum concentration (21.4 hrs) and horses the shortest (1.5 hrs) [20]. In addition to formulation, one important factor that has been suggested to affect the availability of NSAIDS for absorption, alter T_MAX_ and increase half-life in herbivores is binding of the drugs to hay and digesta; however this affect was shown to vary considerably with the specific drug studied [25]. A recent study comparing the pharmacokinetics in ruminant calves fed grass hay versus pre-ruminant calves fed milk replacer and calf starter showed no statistically significant difference in the Tmax or T ½ λ_z_ between ruminant and non-ruminant diets [26]. In addition to anatomic differences and types of feed provided, differing pH of the stomach compartments in ruminant (or ruminant-like) and non-ruminant animals may serve to affect the site, and thus the timing, of meloxicam absorption, as absorption is favored in relatively acidic areas of the gastrointestinal tract [26]. In addition, certain species-specific factors, such as increased drug metabolism, may also play a key role in determining pharmacokinetic differences.

**Table 4:** Comparison of pharmacokinetic parameters of meloxicam in domestic animal species, PO administration

	**PO administration (mean values)**				
**Parameter**	Llamas*	Cattle*	Goats*	Horses^**^	Sheep*
**(Units)**	**(present study)**	[18]	[19]	[20]	[21]
**AUC**_**INF**_(hr*μg/mL)	68.35	164.46	23.24	NR	75.09
**Cl/F (mL/min/kg)**	0.248	0.1	NR	NR	0.220
**C**_**MAX**_**(μg/mL)**	1.31	3.10	0.736	1.73	1.715
**T**_**MAX**_**(hr)**	21.4	11.64	15	3.4	19.0
**T ½ λz (hr)**	22.7	27.54	11.8	NR	15.4
**Vz/F (L/kg)**	0.487	0.242	NR	NR	0.293
**Dose (mg/kg)**	1.0	1.0	0.5	0.6	0.99
**F (%)**	76.0	100.0	79.0	95.6	72.0

NR = not reported.

* Non-compartmental pharmacokinetic analysis.

** Compartmental pharmacokinetic analysis.

AUC_INF_ = area under the curve extrapolated to infinity; Cl/F = Cl per fraction of the dose absorbed; C_MAX_ = maximum plasma concentration; T_MAX;_ = time to C_MAX;_ T ½ λz, T ½_β_ = terminal half-life; Vz/F = Vz per fraction of the dose absorbed; F = fraction of the dose absorbed.

**Table 5:** Comparison of pharmacokinetic parameters of meloxicam in domestic animal species, IV administration

	**IV administration (mean values)**					
**Parameter**	Llamas*	Cattle*	Goats*	Horses^**^	Sheep*	Camels*
**(Units)**	**(present study)**	[18]	[19]	[20]	[21]	[22]
**AUC**_**INF**_(hr*μg/mL)	43.96	82.34	29.74	NR	49.26	346.7
**Cl or Cl**_**T**_**(mL/min/kg)**	0.190	0.1	0.298	0.34	0.169	0.032
**C0(μg/mL)**	6.16	5.93	3.12	NR	4.96	NR
**T ½ λz or T ½**_**β**_**(hr)**	17.4	20.35	10.9	8.54	14.0	40.2
**Vss (L/kg)**	0.235	0.171	0.245	0.12	178.7	0.093
**Dose (mg/kg)**	0.5	0.5	0.5	0.6	0.5	0.6

NR = not reported.

* Non-compartmental pharmacokinetic analysis.

** Compartmental pharmacokinetic analysis.

AUC_INF_ = area under the curve extrapolated to infinity; Cl = plasma clearance; Cl_**T**_ = total body clearance; C0 = Concentration extrapolated to time 0 using log-linear regression of the first two time points; T ½ λz, T ½ _**β**_ = terminal half-life; Vss = volume of distribution at steady state.

In the present study, the half-life of IV meloxicam was 17.4 hrs, which falls in the middle of the range of half-lives observed in other domestic large animal species (from 8.5 hrs in horses to 40.2 hrs in camels) [20,22]. The volume of distribution after IV administration was 0.235 L/kg, which is towards the higher end of the range within the species reported (from 0.093 L/kg in camels to 0.245 L/kg in goats) [19,22] but is still considered to be a relatively low volume of distribution consistently observed with most NSAIDS. The AUC_INF_ in llamas of 46.96 hr*μg/mL was higher than that reported in goats (29.74 hr*μg/mL) [19] but lower than that reported in cattle (82.34 hr*μg/mL) [18] given the same dosage of 0.5 mg/kg. In contrast, however, the AUC_INF_ for camels was markedly higher at 346.7 hr*μg/mL with only a slightly higher dosage of 0.6 mg/kg IV [22]. The extreme variation in AUC_INF_ can be attributed to differences in clearance rates between species which range from 0.032 mL/min/kg in camels [22] to 0.298 mL/min/kg in goats [19], and underscores the need for pharmacokinetic studies in the species of interest to determine accurate estimates of drug concentrations after administration.

The actual therapeutic concentration range in llamas for meloxicam, as well as all other NSAIDS, is unknown. Target ranges for pain relief due to experimentally induced arthritis in horses based on the estimated EC50 value (concentrations that provide 50% of the maximum effect) have been suggested to be >0.2 μg/mL [27] which may allow for cautious extrapolation that this concentration may provide similar therapeutic pain relief benefits in llamas. At an oral dose of 1 mg/kg, concentrations of >0.2 μg/mL are maintained for at least 72 hours, suggesting that a single dosage of oral meloxicam at 1 mg/kg could potentially maintain therapeutic concentrations for pain relief in plasma for 2–3 days in adult llamas. Utilizing the plasma clearance and the oral bioavailability established in this study and a dose of 1 mg/kg, an average concentration at steady state can be estimated to be 2,734 ng/mL with daily dosing, and 1,367 ng/mL when administered every other day. The calculation of the average therapeutic concentrations in plasma during a dose interval in other species from approved maintenance dose and reported clearance values varies from 347 ng/mL in cats, 735 ng/mL in horses to 833 ng/mL in dogs and 1389 ng/mL in humans [19]. This variation makes extrapolations of therapeutic concentrations between species difficult. Additionally, the potential exists for increased risk of adverse effects due to dose accumulation, especially at once daily dosing. To determine optimal dosing and treatment intervals necessary to ensure adequate tissue concentrations for clinical efficacy, further studies combining pharmacodynamics with pharmacokinetics and safety trials in llamas are recommended. In addition, various stressors such as disease, inflammation, pregnancy, and lactation have all been shown to affect the pharmacokinetics of medications [28], thus the data reported here may not reflect exact PK parameters in diseased states. Alternative dosing strategies may also be necessary to achieve appropriate levels of therapy for all targeted outcomes (pain relief, fever reduction, decrease in inflammation, or protection against the effects of endotoxin). Until completion of further studies, it is recommended that dosing be tailored to the clinical response of the individual patient to treatment.

No adverse effects were noted in any animals receiving either oral or IV treatments of meloxicam. To further evaluate the safety of meloxicam administration in healthy llamas, complete blood count and chemistry panels were submitted on all animals prior to and following the second phase of treatment. We were particularly concerned with the effect of treatment on liver and kidney chemistry values, as renal and hepatic toxicity are known complications of NSAID use [17]. As reported in Table 2, all animals consistently displayed a mild hypoalbuminemia and decreased BUN prior to treatment, while five of the six displayed a mild anemia in comparison to the references ranges. These abnormalities may be related to the use of standard reference ranges from Oregon State University Diagnostic Laboratory instead of validation of in-house reference ranges, as differences in instrumentation can lead to variability in results. Decreased BUN and albumin can also be related to feeding of a low protein diet [29]. The level of BUN did increase in a statistically significant manner after treatment with meloxicam, however, the average value was still considered to be lower than the reference range. As these animals were housed at their home farm in between treatment phases, the potential exists for differences in fed protein levels between home farm (pre-treatment) and university (post-treatment) feed sources. Mild anemia and hypoalbuminemia can also be related to intestinal parasitism, which is common in camelids. Fecal samples were taken from 4 of the 6 animals with the lowest hematocrit for McMaster’s Quantitative Fecal Egg Counts; 2 of the 4 were negative for parasite detection, while the other 2 exhibited only very low levels (<100 eggs/gram of Trichostrongyles) of intestinal parasites. This alone is cannot completely rule out current intestinal parasitism as the cause of the mild anemia and hypoproteinemia; in addition, the history of anti-parasiticide administration is unknown in these animals. Therefore, recent on-farm deworming may have affected the results of the fecal exams. The low serum albumin exhibited by the animals in this study also has the potential to affect the pharmacokinetic data of meloxicam as NSAIDS are typically highly bound to plasma proteins [17]; however, there is no evidence available to demonstrate that it impacted the results presented in this study.

One animal exhibited a significantly elevated AST level pre-treatment that had decreased slightly by the post-treatment sample but was still elevated; this animal was otherwise apparently healthy for the entire duration of the study. All other animals had normal AST values pre-treatment; in 2 of the 5 remaining animals, AST levels were increased above pre-treatment levels in post-treatment samples, however, only 1 additional animal exhibited AST levels greater than the reference range post-treatment. The difference in AST between pre- and post-treatment blood samples was not considered to be statistically significant. One animal exhibited elevated GGT following treatment, but overall change in GGT for all animals was not statistically significant. Statistically significant increases were seen in chloride, while statistically significant decreases were noted with bicarbonate, non-esterified fatty acids (NEFAs) and creatinine. However, all values for these parameters were still within normal references ranges and thus the differences are not considered to be biologically significant. Further studies involving multiple dosages and increased duration of therapy would be warranted to verify that there are no negative effects on either kidney or liver function with prolonged therapy.

In addition to risk of nephrotoxicity and hepatotoxicity, NSAID usage has been commonly recognized as a risk factor for gastric ulceration in many species, including humans, cats, dogs and horses, and it has been suggested as a potential risk factor for ulceration of the third compartment of the camelid stomach (C3) [30,31]. In llamas, C3 ulcers are a commonly encountered gastrointestinal disorder, however, there is a lack of scientific data detailing the pathogenesis of ulcer formation in this species [31,32]. It has been suggested that stress, especially related to disease and chronic disorders or isolation from herd mates, may play a role in the development of ulcerations in llamas [31]. Non-selective NSAIDS decrease the production of prostaglandins thought to be important in maintaining mucosal blood flow and mucous secretion in mammals; it is a combination of this and other effects that is thought to lead to gastric ulceration with NSAID use [33]. Previous studies involving daily intramuscular administration of 1.1 mg/kg flunixin meglumine have demonstrated a lack of statistically significant gastric pH reduction and ulcer formation in llamas, which led the authors of that study to suggest that NSAIDS may not exert similar ulcerogenic effects in this species [34]. However, recent literature reviews on NSAID-induced gastropathy do not suggest direct reduction of pH as an inciting cause of ulceration [17,33,35]. Thus, with a lack of strong scientific data to suggest that NSAIDS do not exert an ulcerogenic effect in llamas, it may be prudent to utilize NSAIDS such as meloxicam that have exhibited improved GI safety profiles in other species [36].

Although reports vary, extensive work in humans suggests that while not completely free from GI-related side effects, there is a greater GI safety profile for COX-2 selective inhibitors in comparison to non-selective inhibitors [17,36-38]. Meloxicam is generally considered to be a COX-2 selective drug; however, studies have shown that there can be much variability between species in the preference of inhibition of cyclooxygenase-1 versus cyclooxygenase-2 for a given drug. In humans, meloxicam demonstrates a significant and long lasting preference for inhibition of COX-2 while having much less activity against COX-1 [39]. In dogs, in vitro assays suggest that meloxicam ranges from 3.7 to 12 times more effective at inhibiting COX-2 versus COX-1 [40-42]. However, in cats, both in vitro and ex vivo studies have shown a ratio of only 2.7 in regards to selectivity of COX-2 inhibition over COX-1 [43]. Early meloxicam research in horses also suggests that even though present, suppression of COX-1 is reversible, and COX-2 inhibition is prolonged at sites of inflammation [44]. Therefore, meloxicam may offer some selective benefit in llamas of COX-2 inhibition over the non-selective NSAIDS, but further studies are warranted to determine to what extent it can be considered COX-2 selective in this species.

## Conclusions

The results of the present study suggest that oral administration of meloxicam may offer a practical and long-acting option for NSAID administration for the relief of pain and inflammation in llamas. The pharmacokinetic profile described in this study supports further research including pharmacodynamic studies and efficacy trials to best determine appropriate dosage and treatment intervals for certain disorders. Additional studies evaluating response to multi-day therapy regimens are also warranted to fully assess the pharmacokinetic and safety profile of this COX-2 selective NSAID in llamas.

## Methods

This study was approved by the Institutional Animal Care and Use Committee at Iowa State University (ISU) (Protocol # 10-11-7239-V).

### Animals and housing

Six healthy adult llamas ranging in age from 2 to 14 years were used for this study. Mean (± standard deviation) weights at first and second treatment administrations were 144.0 ± 18.31 kg and 149.8 ± 18.88 kg respectively. Weights for dose calculation were determined by weighing the llamas 24 hours prior to each treatment administration.

Study animals were placed in group housing at the university for 1 day prior to each phase of the study and returned to their home farm in between study phases. Housing consisted of an indoor stall with rubber mat flooring at the Iowa State University Lloyd Veterinary Medicine Center. All llamas were fed grass hay and water *ad libitum* throughout the experiment. Temperature, pulse, respiration, urination, defecation, attitude and appetite were monitored throughout each study period.

### Experimental design

A cross over study design was used with randomized assignment of llamas to one of 2 dosing regimens. The observed washout period between treatment administrations was 11 days.

Approximately 18 hours prior to study commencement, llamas were restrained for intravenous catheter placement. Llamas that received IV meloxicam were fit with two catheters. One catheter was designated for drug administration only and the other solely for blood sample collection. The area over the cranial portion of the jugular vein was clipped and disinfected using alternating 70% isopropyl alcohol and chlorhexidine soaked 4x4 gauze. For ease of catheter placement, all animals were sedated with approximately 0.1 mg/kg of xylazine IV (Anased® injection, 100 mg/mL, Lloyd Laboratories, Shenandoah, IA). The catheter site was infiltrated with 2% lidocaine injection, 1 ml SQ (Hospira Inc, Lake Forest, IL) prior to placement of catheter and a small stab incision was made through the skin at the preferred site for catheter placement. A 14 G x 140 mm catheter (Abbocath-T®, Hospira, Slingo, Ireland) was placed on the side designated for blood sample collection (right jugular), while a 16 G x 51 mm catheter (Abbocath-T®, Hospira, Slingo, Ireland) was placed on the side designated for drug administration (left jugular). If unable to place the 14 G x 140 mm catheter due to the presence of a valve in the jugular vein, the 16 G x 51 mm catheter was alternatively placed for the blood draw catheter. All catheters were sutured to the skin using 2–0 nylon suture (Ethilon®, Ethicon Inc, San Lorento, Puerto Rico) and a 15.2 cm high flow extension set with reflux valve (MILA International, Florence, KY) was added to the catheter to prevent backflow of air and aid in ease of blood sample collection. Catheter patency was maintained using heparin saline flush containing 2 USP units heparin sodium/mL saline (Heparin Sodium Injection, B Braun Medical, Irvin, CA).

Each llama was subjected to the following two treatments (n = 3 llama/treatment/period);

1) Intravenous (IV) injection of 0.5 mg/kg of meloxicam (Metacam® 5 mg/mL solution for injection (NADA 141–219), Boehringer Ingelheim Vetmedica, Inc. St Joseph, MO; Lot # 2066180) administered as a bolus in the jugular vein using a designated catheter. The catheter was flushed with 6 mL of heparin-saline and removed immediately after flushing.

2) Oral (PO) meloxicam was administered at 1 mg/kg (Meloxicam tablets 15 mg (NDC 0378-1089-01), Mylan Pharmaceuticals, Morgantown, WV; Lot # 3025543). Tablets were dissolved in 50 mL of water within 60 minutes of administration by stomach tube. The stomach tube was flushed with 500 mL of water prior to removal.

The drug doses were selected based on the study designed used in previous publications that explored the oral bioavailability of meloxicam in ruminants where it was expected that drug absorption from the gastrointestinal tract may be diminished [18,21]. The IV dose was rounded to the nearest 1.0 mL and administered using a 20 mL syringe. The oral dose was rounded to the nearest whole tablet and administered in water with a 60 mL catheter tip syringe.

Llamas were manually restrained with a halter and lead rope for blood collection. In the llamas receiving IV meloxicam, approximately 6 mL of blood was collected at 0, 3, 6, 10, 20, 40 minutes and 1, 3, 6, 12, 24, 48, 72 and 96 hours after dosing. Llamas receiving oral meloxicam were blood sampled at 0 and 30 minutes and 1, 2, 4, 6, 8, 10, 12, 24, 48, 72 and 96 hours after administration. Blood was drawn into a collection syringe and immediately transferred to lithium heparin vacutainer tubes (HemoGARD®, BD Diagnostics, Franklin Lakes, NJ). Samples were stored on ice prior to centrifugation at 5°C for 10 minutes at 1,500 x g within 120 minutes of collection. Plasma was then pipetted to cryovials and frozen at −70°C until analysis.

Blood samples from prior to initiation of the second phase of the trial (t = 0 min) and at 72 hrs post treatment were also collected into serum (BD Vacutainer®, BD Diagnostics, Franklin Lakes, NJ) and EDTA (Monoject^™^ 15% EDTA(K3), Tyco Healthcare Group, Mansfield MA) tubes and submitted within 1 hr to the Iowa State Lloyd Veterinary Medical Center Clinical Pathology Department for a complete blood count (CBC) and blood chemistry to assess the effect of meloxicam treatment on hematologic parameters in llamas.

A 2-way ANOVA was used to compare hematological values before treatment [baseline] vs. after treatment as well as treatment type [oral vs intravenous] in the second phase of the study. When the overall treatment effect was significant, a posthoc Tukey test was used to determine whether there was a greater change for that variable before and after exposure to each treatment. Statistical significance was set *a priori* at values of P ≤ 0.05. All statistical analyses were performed by use of statistical software (JMP 9.0, SAS Institute, Cary, NC).

### Plasma drug analysis

Plasma concentrations of meloxicam were determined using high-pressure liquid chromatography (Surveyor MS Pump and Autosampler, Thermo Scientific, San Jose, CA, USA) with mass spectrometry detection (TSQ Quantum Discovery MAX, Thermo Scientific, San Jose, CA, USA). Plasma samples, plasma spikes, and plasma QC samples, 0.200 mL, were treated with 30% perchloric acid (20 μL) after addition of 40 ng/mL of the internal standard piroxicam. The samples were vortexed for 5 seconds and centrifuged for 20 minutes at 2,500 × g to sediment the precipitate. A portion of supernatant, 80 μL, was transferred to an injection vial fitted with a glass insert containing 120 μL of 1.9% ammonium hydroxide in 25% aqueous acetonitrile. The injection volume was set to 12.5 μL. The mobile phases consisted of A: 0.1% formic acid in water and B: 0.1% formic acid in acetonitrile at a flow rate of 0.225 mL/min. The mobile phase began at 25% B with a linear gradient to 95% B at 7 minutes, which was maintained for 1.5 minutes, followed by re-equilibration to 25% B. Separation was achieved with a solid-core C18 column (KinetexXB -C18, 100 mm × 2.1 mm, 2.6 μm particles, Phenomenex, Torrance, CA, USA) maintained at 40°C. Piroxicam eluted at 4.85 minutes and meloxicam at 5.95 minutes. Four SRM transitions were monitored for meloxicam and three SRM transitions were used with the internal standard, piroxicam. The quantifying ions for meloxicam were 72.99, 88.01, 114.99, and 140.98 m/z and 77.97, 94.98, and 120.98 m/z for piroxicam. Sequences consisting of plasma blanks, calibration spikes, QC samples, and llama plasma samples were batch processed with a processing method developed in the Xcalibur software (Thermo Scientific, San Jose, CA, USA). The processing method automatically identified and integrated each peak in each sample and calculated the calibration curve based on a weighted (1/X) linear fit. Plasma concentrations of meloxicam in unknown samples were calculated by the Xcalibur software based on the calibration curve. Results were then viewed in the Quan Browser portion of the Xcalibur software. The standard curve in llama plasma was linear from 0.005 to 10.0 μg/mL. The coefficient of determination (R squared) exceeded 0.995 and all measured values were within 15% of the actual values with most of the values less than 5% difference from the actual values. The accuracy of the assay for meloxicam in llama plasma was 99 ± 3% of the actual concentration while the coefficient of variation was 5% determined on 4 sets of replicates for each of the following concentrations: 0.015, 0.15, and 1.5 μg/mL. The limit of quantitation (LOQ) for this assay was determined to be 0.005 ug/mL, while the limit of detection (LOD) was 10-fold lower than that at 0.0005 ug/mL.

### Pharmacokinetic analysis

Pharmacokinetic analyses were performed with computer software (WinNonlin 5.2, Pharsight Corporation, Mountain View, CA, USA) using noncompartmental methods. The variables calculated included the area under the curve from time 0 to infinity (AUC_INF_) using the linear trapezoidal rule, area under the first moment curve from time 0 to infinity (AUMC_INF_), plasma clearance (Cl), plasma clearance per fraction of the dose absorbed (Cl/F), apparent volume of distribution at steady state (Vss), apparent volume of distribution of the area (Vz), apparent volume of distribution of the area per fraction of the dose absorbed (Vz/F), first-order rate constant (λ_z_), terminal half-life (T_½_ λ_z_), and mean residence time extrapolated to infinity (MRT). The maximum serum concentration (C_MAX_) and time to maximum serum concentration (T_MAX_) were determined directly from the data. The concentration at time 0 (C0) was calculated by log-linear regression using the first two time points after IV administration. The mean absorption time (MAT) was calculated by subtracting the IV MRT from the PO MRT. The fraction of the dose absorbed (F) for oral meloxicam was determined by dividing the AUC_INF_ per dose after oral administration by the AUC_INF_ per dose after IV administration.

In order to determine if the outlier data observed in one animal due to an error in bodyweight determination prior to study commencement significantly affected the mean calculated pharmacokinetic parameters, a Wilcoxon-rank sum test was used to compare non-normally distributed data (T_MAX_, MAT) and a Students t-test was used for normally distributed data including and excluding the outlier. Statistical significance was designated a priori as p < 0.05. All statistical analyses were performed by use of statistical software (JMP 9.0, SAS Institute, Cary, NC).

## Abbreviations

AUC extrapolated, Percent of the AUC extrapolated; AUCINF, Area under the curve extrapolated to infinity; Cl, Plasma clearance; Cl/F, Cl per fraction of the dose absorbed; C0, Concentration extrapolated to time 0 using log-linear regression of the first two time points; CMAX, Maximum plasma concentration; TMAX, Time to CMAX; T ½ λz, Terminal half-life; λz, Terminal rate constant; MRT, Mean residence time extrapolated to infinity; Vss, Volume of distribution at steady state; Vz, Volume of distribution, area method; Vz/F, Vz per fraction of the dose absorbed; MAT, Mean absorption time; F, Fraction of the dose absorbed; EC50, Concentration necessary to reach 50% of estimated maximal effect; COX, Cyclo-oxygenase; HPLC-MS, High pressure liquid chromatography and mass spectrometry detection; PK, Pharmacokinetic; NSAIDS, Non-steroidal anti-inflammatory drugs; IV, Intravenous; PO, Per os (by mouth); EU, European Union; BUN, Blood urea nitrogen; AST, Aspartate aminotrasferase; GGT, Gamma-glutamyl transpeptidase; NEFAS, Non-esterified fatty acids; C3, Third compartment of the camelid stomach; GI, Gastrointestinal; SQ, Subcutaneous; EDTA, Ethylene diamine tetra-acetic acid; CBC, Complete blood count; ANOVA, Analysis of variance; QC, Quality control.

## Competing interests

The authors state that there are no competing interests related to the present study**.**

Dr. Coetzee has been a consultant for Intervet-Schering Plough Animal Health, Boehringer-Ingelheim Vetmedica and Norbrook Laboratories Ltd.

Dr. KuKanich has been a consultant for Bayer Animal Health, Central Life Sciences, Pfizer Animal Health, and Procyon Pharmaceuticals.

## Authors’ contributions

JFC conceived the study, participated in the design and coordination, performed the pharmacokinetic analysis and aided in drafting the manuscript. AJK participated in the design and coordination and drafted the manuscript. PJP participated in the design and coordination, and aided in drafting the manuscript. JAS participated in the study design and aided in drafting the manuscript. BK assisted with the pharmacokinetic analysis. LLL assisted in the coordination, data compilation and sample processing. LWW performed the plasma drug analysis on all samples. All authors read and approved the final manuscript.
